# Effects of Positive and Negative Experiences on Cooperative Behavior: The Role of Sharedness

**DOI:** 10.3390/ijerph20010636

**Published:** 2022-12-30

**Authors:** Fangzhu Qi, Wei Wang, Minghui Wang, Yongfang Liu

**Affiliations:** 1 Institute of Psychology and Behavior, Henan University, Kaifeng 475004, China; 2 School of Psychology and Cognitive Science, East China Normal University, Shanghai 200062, China

**Keywords:** positive experience, negative experience, sharedness, shared experience, cooperative behavior

## Abstract

Cooperation is a fundamental ingredient of society. However, research on the effects of positive and negative experiences on cooperation remain largely inconsistent. Therefore, through two experiments, the present study examined the effects of positive and negative experiences on cooperative behavior, and the moderation effect of sharedness on this relationship. In Study 1, we directly compared positive and negative experiences in the same context. Seventy-four students participated the experiment (*M*_age_ = 19.88, *SD*_age_ = 2.21). Results showed that participants reported higher levels of cooperative behavior in negative experiences than in positive experiences. In Study 2, we examined the moderation effect of sharedness on the relationship between positive/negative experiences and cooperative behavior. The result of the experiments involving 126 participants (*M*_age_ = 19.53, *SD*_age_ = 1.14) showed a significant interaction effect between positive/negative experiences and sharedness on cooperative behavior. Participants exhibited higher level of cooperative behavior in shared negative experiences than in shared positive experiences, while there were no significant differences in cooperative behavior between unshared negative and positive experiences. These results suggested that shared negative experiences could facilitate cooperative behavior.

## 1. Introduction

Cooperation is an essential component of society. It generates benefits at the group level and costs at individual level, and is the choice to give up the possibility of maximizing individual interests to benefit group interests [1]. Researchers indicated that cooperation is the third evolutionary path, besides gene mutation and natural selection, which are the driving forces of human evolution and development [2]. Whether to cooperate or not is not simply a decision about a mathematical calculation of the amount of economic interest. It can be influenced by many social and psychological factors, in which positive and negative experiences play important roles in people’s decision-making to cooperate [3]. Meanwhile, positive or negative experiences may have stronger psychological effects than the neutral experience. For instance, individuals have more profound memories of positive or negative events [4,5], and prefer to share those experiences with others [6,7]. However, the underlying mechanism and effect of positive and negative experiences on cooperation remain unclear. Some studies indicated that positive experiences could promote cooperation [8], while others found that negative experiences could promote it [9].

Previous studies generally supported that positive experiences strengthened cooperation, while negative experiences ruined it [10]. This might be due to several reasons. First, prior studies might have solely examined the effect of positive [11] or negative experiences [12] and compared them with neutral experiences. Thus, there may be a lack of perspective in directly comparing the effects of positive and negative experiences. Furthermore, studies focusing on the effect of positive experiences tended to pay attention to its positive effect, while those on the effect of negative experiences tended to pay attention to its negative effect [13]. Additionally, studies that showed negative experiences could decrease cooperative behavior usually involved samples with psychological abnormalities, such as post-traumatic stress disorder [14], while those on positive experiences rarely involved people with psychological abnormalities. Therefore, samples of these studies cannot be compared directly. Overall, there is insufficient research which directly compares the effects of positive and negative experiences on cooperative behavior in the same context. Therefore, the fist experiment of the present study aimed to directly compare the effects of positive and negative experiences in the same context.

Furthermore, an increasing number of recent studies began to indicate that negative experiences could benefit cooperation. Prior studies showed negative experiences could promote interpersonal relationships, such as empathy [15], trust [16], social bonding [17], helping behavior [18], and cooperative behavior [19]. These studies were conducted in a condition in which negative experiences were shared with others. In other words, negative experiences promoted cooperation in a shared condition [17], while studies showing positive experiences affecting cooperation did not manipulate the condition of sharedness. Therefore, it is unclear whether the conclusion that negative experiences promote cooperation is drawn from “shared negative experience” or “negative experience”. Although an increasing amount of evidence shows painful experiences (a kind of negative experience) promoted cooperation [16,17], studies also found pain could increase aggressive behavior [12]. These inconsistent results suggest the possibility that moderating variables exist in the relationship between positive/negative experiences and cooperative behavior. To our knowledge, only one study provided explicit evidence that sharedness moderated the effect of pain on cooperative behavior [20]. Additionally, prior studies also showed that a crucial function of shared experiences is to facilitate interpersonal connection and closeness [21], and that it acts as an important antecedent of social decision-making [22]. Thus, the second experiment of the present study aimed to examine the moderation effect of sharedness on the relationship between positive/negative experiences and cooperative behavior.

Above all, we will directly compare the effects of positive and negative experiences on cooperation, and further introduce sharedness to investigate the boundary condition of the effects. Through these two studies, we aim to clarify how positive/negative experiences affect cooperation and improve the understanding of laws of cooperation. In previous studies on the effects of the positive experiences or negative experiences on cooperation, researchers usually invited university students as participants [12,16,17]. To be consistent with previous studies, university students were selected as participants in the present study.

## 2. Study 1

Study 1 aimed to examine the effects of positive/negative experience on cooperative behavior in a social dilemma. We directly compared negative experience with positive experience in the same context.

### 2.1. Materials and Methods

#### 2.1.1. Participants

Participants were 74 undergraduate students recruited at a college in central China (*N*_women_ = 37, *M*_age_ = 19.88, *SD*_age_ = 2.21). The participants were randomly allocated to different groups, with three or four participants in each group (the average number of participants in the groups was 3.59, with a median of 4). Participants were not involved in similar experiments before. Informed consent was obtained from the participants before the experiment was conducted. After the experiment, each participant was paid CNY 20.

#### 2.1.2. Design

The experiment had a single factor between-subjects design (two conditions: positive experience vs. negative experience). The dependent variable was cooperation level measured by using the economic game.

#### 2.1.3. Materials

The food preference paradigm was used to manipulate the valence of experience [17,23]. Participants were told that the experiment was on consumer preferences, and that they needed to evaluate the preference of the food. Participants in the positive experience group were given rock candy, while those in the negative experience group were given fresh and clean pepper. They were instructed to eat as much as possible of the food sample.

The following is an example of the instructions to the negative experience group:

*…Your task is to build a food preference norm.*

*…The orange box on the table contains the food you need to taste, that is, a certain type of pepper. Please taste and chew the pepper carefully as much as possible and spit it out when you feel unbearable (you have to taste one pepper for at least in 90 s). You can spit the food residue into a paper cup, and continue to taste a new pepper. When you hear the instruction “task start”, please start tasting the food immediately. Don’t hesitate and wait.*

After tasting the pepper or rock candy, participants needed to answer two questions for manipulation check: “When you tasted the food, how strong was the pain you experienced?”, “When you tasted the food, how unpleasant was the pain you experienced?” Each item was rated on a 10-point Likert scale ranging from 1 (not at all) to 10 (very much).

The cooperative behavior was measured by the economic game, which was used to assess how participants weighed individual and group interests in previous studies [17,23]. The more favorable the choice was to the group interest, the more cooperative the participant was. The specific rules of the economic game were as follows. A group of participants played the game in which each participant would choose a number from one to seven (for the full payoff schedule, see Table 1). The higher the lowest number in the group, the higher the economic outcomes for the group. When participants chose the same numbers, the payoff for each participant was equal. When participants chose different numbers, the higher the chosen number, the lower the payoff received. Thus, choosing “7” represented the most cooperative option because it maximized the potential payoff for the group, but the participants had the greatest risk of receiving the lowest payoff if others chose a number lower than “7”. Choosing “1” represented the least cooperative option because it ensured a moderate-sized payoff for the participant (CNY 4.20; compared to the maximum potential individual payoff CNY 7.80 and the minimum potential individual payoff CNY 0.60; CNY 4.20 was a moderate-sized individual payoff) but minimized the potential payoff for the group. Thus, the higher the number a participant chose, the more cooperative he/she was. The game was conducted for six rounds and the average number that one chose was indexed as the cooperation level. To minimize interactive interference, participants were instructed that the game would be conducted for several rounds and that their final payoff would be determined according to a random round.

#### 2.1.4. Procedure

A group of participants arrived at the same laboratory and completed the informed consent. They were randomly assigned to the positive/negative experience conditions. Experimenters instructed them to finish the food preference task and the economic game. After the economic game, participants were thanked and paid.

### 2.2. Results

#### 2.2.1. Manipulation Check

Independent sample *t*-tests were conducted to examine whether the manipulation succeeded. The results showed that the participants reported stronger pain in the negative experience condition (*M* = 7.57, *SD* = 1.24) than in the positive experience condition (*M* = 1.43, *SD* = 0.69), *t* (72) = 26.36, *p* < 0.001, *d* = 6.12. Additionally, they reported higher unpleasant experience in the negative condition (*M* = 6.81, *SD* = 2.38) than in the positive condition (*M* = 1.86, *SD* = 1.23), *t* (72) = 11.24, *p* < 0.001, *d* = 2.61. These results suggested the manipulation of positive/negative experiences was successful.

#### 2.2.2. Effects of Positive/Negative Experience on Cooperative Behavior

**Figure 1:**
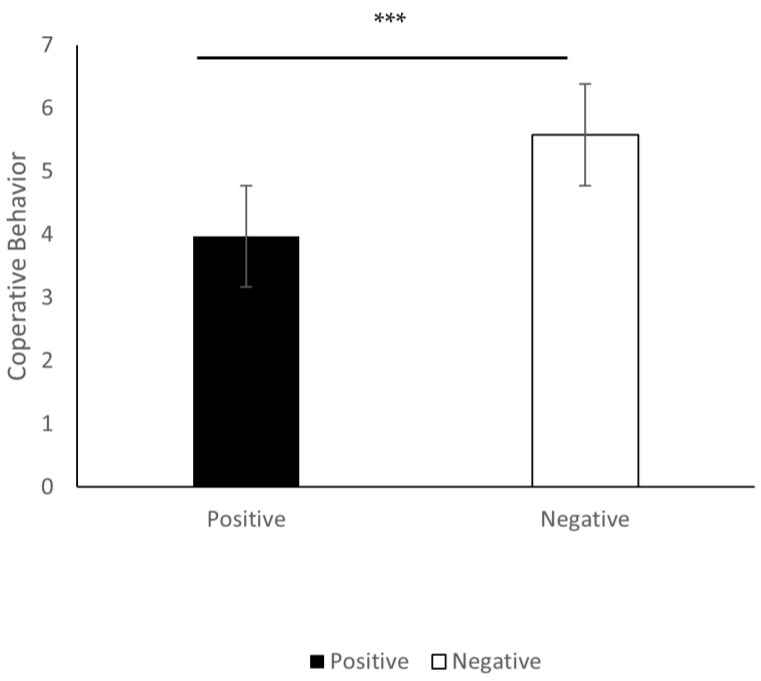
Effects of positive/negative experience on cooperation. *** *p* < 0.001; error bar represents standard error.

The average of the numbers chosen by the participants in Experiment 1 was indexed as the indicator of cooperative behavior. Analysis of variance (ANOVA is a statistical model and data analysis tool used to analyze the differences among means) was conducted with cooperative behavior as the dependent variable and positive/negative experience as the independent variable; the results are shown in Figure 1. It showed that the main effect of positive/negative experience was significant, *F* (1, 72) = 35.97, *p* < 0.001, $\eta_{p}^{2}$ = 0.33. Participants who tasted pepper (*M* = 5.58, *SD* = 1.08) chose a significantly higher number than those who tasted rock candy (*M* = 3.97, *SD* = 1.23), indicating that participants with a negative experience would engage in more cooperative behavior.

## 3. Study 2

Based on the results of Study 1, Study 2 examined the moderation effect of sharedness on the relationship between positive/negative experience and cooperative behavior. Additionally, Study 2 adopted another classic social dilemma paradigm—the public goods game—to examine the robustness of the effect of positive and negative experiences on cooperative behavior.

### 3.1. Materials and Methods

#### 3.1.1. Participants

Participants were 126 undergraduate students recruited at a college in central China (*N*_women_ = 126, *M*_age_ = 19.53, *SD*_age_ = 1.14). They were randomly allocated to different groups, with three or four participants in each group (the average number of participants in groups was 3.15, with a median of 3). Participants were not involved in similar experiments before. Informed consent was obtained from the participants before the experiment was conducted. After the experiment, each participant was paid CNY 20.

#### 3.1.2. Design

The experiment had a 2 (positive/negative experience: positive vs. negative) × 2 (sharedness: shared vs. unshared) between-subjects design. The dependent variable was the cooperation level measured through the public goods game.

#### 3.1.3. Materials

Manipulation of positive/negative experiences was same as Study 1.

Participants in the shared group were told “You need complete the same task with other members in your group. The content and materials are exactly the same, and the materials needed in the task are placed on the table”. Those in the unshared group were told “You need complete a different task compared with other members in your group. The content and materials are different from others, and the materials needed in the task are placed on the table”. In reality, in both conditions, participants in each group tasted the same food (i.e., pepper or rock candy). Although the group of participants were in the same room, the baffles ensured they could not see what other participants were doing. During the experiment, participants were asked to wear headphones to minimize external interference.

After tasting the pepper or rock candy, participants were asked to answer three questions for manipulation check. Two questions for manipulation of positive/negative experience were the same as in Study 1. One question for manipulation of sharedness was: “Do you think the content of your task was the same as that of other participants in the last session?” The item was rated on a 10-point Likert scale ranging from 1 (completely different) to 10 (completely the same).

Cooperative behavior was measured by a no-feedback public goods game (PGG). Each participant had a personal account with CNY 20 initially. Additionally, there was a public account which had CNY 0 initially (to increase the authenticity of the experiment and improve the participants’ involvement, real money was used). In each round, a participant needed to make an allocation and decide the money to keep in their personal account and to invest into the public account. The money kept in the personal account would still belong to the individual, while the money received in the public account would be doubled and then distributed equally to each participant. It should be noted that each participant in the PGG would benefit equally from the public account, regardless of whether they invested, or the amount they invested into the public account. The final payoff for each participant consisted of the money remaining in the personal account and that obtained from the public account. Thus, the final payoff for each participant was determined by their own and other participants’ allocation decisions. More investment into the public account represented a higher cooperation level. A participant’s final payoff (Pi) can be expressed by the following formula:

$$P_{i}=20-x_{i}+(2\overset{n}{\underset{i=1}{\sum }}x_{i})/n$$

Note: n represents the total number of participants in the PGG, and x_i_ represents the investment amount of the ith participant, which is ranged from 0 to 20.

#### 3.1.4. Procedure

Same as in Study 1.

### 3.2. Results

#### 3.2.1. Manipulation Check

Independent sample *t*-tests were conducted to examine whether the manipulation succeeded. Results showed that the participants reported stronger pain in the negative condition (*M* = 6.98, *SD* = 2.07) than in the positive condition (*M* = 1.24, *SD* = 1.02), *t* (124) = 19.63, *p* < 0.001, *d* = 3.52. Additionally, participants reported higher unpleasant experience in the negative condition (*M* = 6.73, *SD* = 2.50) than in the positive condition (*M* = 1.27, *SD* = 0.55). These results suggested that the manipulation of positive/negative experience was successful. For the manipulation of sharedness, participants in the shared group (*M* = 6.68, *SD* = 2.02) reported perceiving a higher similarity of the tasks with other participants than those in the unshared group (*M* = 5.68, *SD* = 2.26), *t* (124) = −2.62, *p* = 0.010, *d* = 0.47. These results suggested the manipulation of sharedness was successful.

#### 3.2.2. Effects of Positive/Negative Experience on Cooperative Behavior

**Figure 2:**
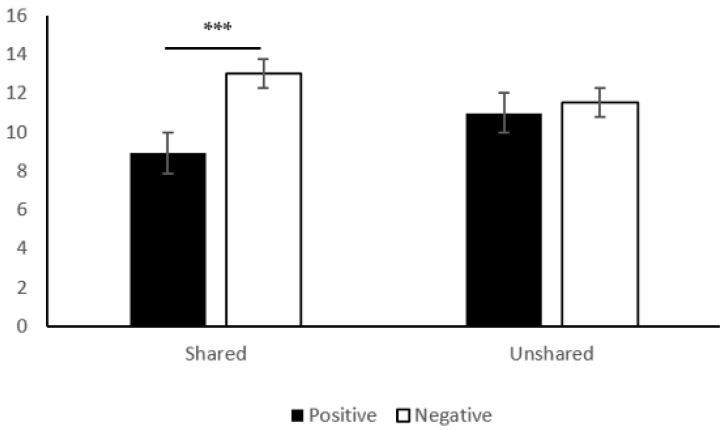
Effects of positive/negative and shared/unshared experience on cooperation. *** *p* < 0.001; error bar represents standard error.

The average amount of participants’ investment in Study 2 was indexed as the indicator of cooperative behavior. A two-way between-subject ANOVA was conducted with cooperative behavior as the dependent variable and positive/negative experience and sharedness as the independent variables. Results are shown in Figure 2. It showed that the main effect of positive/negative experience was significant, *F* (1, 122) = 10.94, *p* = 0.001, $\eta_{p}^{2}$ = 0.08. The investment amount of the participants who tasted pepper (*M* = 12.26, *SD* = 4.06) was significantly higher than that of the participants who tasted rock candy (*M* = 9.92, *SD* = 4.00), indicating that participants with the negative experience would engage in more cooperative behavior. The main effect of sharedness was not significant, *F* (1, 124) = 0.19, *p* = 0.662, $\eta_{p}^{2}$ = 0.00.

The interaction effect (an interaction effect refers to the role of a variable in an estimated model, and its effect on the dependent variable; a variable that has an interaction effect will have a different effect on the dependent variable, depending on the level of some third variable) between positive/negative experience and sharedness was significant (*F* (1, 122) = 6.34, *p* = 0.013, $\eta_{p}^{2}$ = 0.05). The results of simple effect analysis are shown in Figure 2. In the shared condition, participants in the negative condition invested more (*M* = 13.02, *SD* = 3.73) than those in the positive condition (*M* = 8.91, *SD* = 3.32), *F* (1, 122) = 16.98, *p* < 0.001, $\eta_{p}^{2}$ = 0.12. In the unshared condition, there was no significant difference in the investment amount between the participants in negative (*M* = 11.55, *SD* = 4.29) and positive conditions (*M* = 10.99, *SD* = 4.42), *F* (1, 122) = 0.31, *p* = 0.577, $\eta_{p}^{2}$ = 0.00, indicating that when participant did not share experience with others, their positive/negative experience did not influence the level of cooperative behavior.

## 4. Discussion

The present research examined the effects and boundary condition of positive and negative experiences on cooperation. In previous studies, whether positive or negative experiences promoted cooperation remained unclear. Study 1 directly compared the effects of positive experience and negative experience on cooperation, and Study 2 introduced sharedness to further explore the boundary condition of positive/negative experiences on cooperation. Specifically, Study 1 compared the effects of positive and negative experiences on cooperative behavior in the same situation, and the results showed that people were more cooperative in the negative experience condition than in the positive experience condition. Study 2 examined the boundary condition of Study 1, and the results showed that the interaction effect between positive/negative experience and sharedness on cooperative behavior was significant. In other words, when individuals experienced the same event, they showed more cooperative behavior in the negative condition than in the positive condition. However, when individuals experienced different events, there was no significant difference in cooperative behavior between positive and negative conditions. This result was consistent with Study 1, in which participants tasted pepper or candy in the same room, and they could see that other participants were experiencing the same.

The theoretical contribution of this study is that it reconciled the conflicts between the effects of positive and negative experiences on cooperation. The results of the two experiments provide new evidence that positive/negative experience does not directly affect cooperation, which is consistent with previous studies. Researchers compared cooperative behavior (using the chicken game) in happiness–sadness and security–insecurity conditions; participants’ mood states could not predict the cooperative behavior in both cases [24]. The conclusion of positive/negative experience could directly predict cooperative behavior was usually based on the general deduction of different experimental results, rather than a direct comparison between positive and negative experiences in the same situation. Our study directly compared the effects of positive and negative experiences on cooperative behavior and provided evidence on the moderating variables. Regarding the effect on promoting cooperation, some researchers believe positive experiences perform better than negative experiences [10], while others believe negative experience may have stronger effect on cooperative behavior [25]. The conflicts reflect that the effects of positive/negative experience on cooperative behavior may be moderated by other variables. Our study confirmed the moderation effect of sharedness of experience on this relationship.

The moderation effect of sharedness on the relationship between positive/negative experiences and cooperative behavior was consistent with prior studies. For instance, a prior study indicated that participants showed more cooperative behavior in the shared ice water condition than in the shared warm water condition [17]. Then some researchers found that participants who received capsaicin stimulation together showed more trust than those who received hand cream stimulation together [16]. Recently, researchers found that, in addition to shared physical pain, shared social exclusion experience could also promote the level of cooperation [25]. Therefore, this study provided new evidence to the literature that reported shared negative experiences could work as social glue.

According to the social function theory of affect, emotions evolved to help people adapt to the changing environment, therefore, the effects of feeling are contextual [26,27]. Feelings could trigger psychosocial reactions based on their signals and social meanings. In the shared condition, individuals rely on the similarity clues to blur the boundaries between individuals and clarify the boundaries between groups, especially when the original connection and interaction between individuals are weak or non-existent [27]. In the present study, participants were unfamiliar with each other and the group boundary was vague. The shared experience could provide the basis of groups. The shared negative experience could convey a signal that the collective interests were threatened and the group was in an unsafe situation, which triggered stronger needs to belong, resulting in support to the group [25]. Cooperation could create advantage for groups to survive in high-risk collective activities and succeed in group conflicts. It is a conducive way to protect collective interests during social difficulties. In the shared negative experience condition (eating chili peppers together), individuals gave priority to protect the collective interest and showed more cooperative behaviors for balancing the self–others (collective) interest. This result is consistent with the finding that collective threats have positive effect on people’s donation intention, especially for those who experience the same collective threats [28]. Our results provided new evidence to support the following point: from the perspective of evolution, if members are more cooperative when experiencing co-existence anxiety (such as physical pain and other events that have negative effects on health), they are more likely to win in the subsequent inter-group competition [29]. Shared positive experiences usually mean that the current situation is safe and harmless for the group. In this condition (eating candy together), individuals might feel safe and decrease necessity of safeguarding the collective interest. It was not necessary for them to pay high costs to adopt collective behavior [30]. Above all, our findings indicated that shared and unshared experiences had different psychological meanings, and then affected cooperative behavior differently. Sharedness could moderate the effects of various experiences on cooperation. Future studies on effects of various experiences on cooperation could take sharedness into account. Our findings showed shared positive and negative experiences induced different levels of cooperation, indicating that different kinds of shared experiences had unequal effects. When exploring the effects of shared experiences on cooperation, it is important to consider what the experiences are.

Although our findings improved the current understanding of the effects of shared experience on cooperative behavior, there are some limitations. Our findings showed that a shared negative experience induced more cooperative behavior than a shared positive experience. However, it is unclear whether a shared negative experience could promote, or a shared positive experience could decrease, cooperative behavior. In future research, neutral experiences should be compared with positive/negative experiences. Another limitation is that all the participants in Study 2 were women. Although gender equivalence between groups was ensured, whether the effect obtained in this study can be generalized to the whole population remains to be discussed.

Consistent with previous studies, the present study also manipulated the positive/negative experiences using physical experiences [16,17,20,23]. However, research should not be limited to physical experiences. Physical experiences (e.g., physical pain) implicitly play a similar role as social experiences (e.g., social pain) in affecting individuals’ behavior [12]. Positive/negative experiences also include social experiences, such as success/failure and social acceptance/exclusion, which should be explored in future research.

In social dilemmas, individuals have to weigh between their own interest and others’ interests. Thus, as an important representation of the self–other relationship [31], psychological distance is a crucial factor to clarify the law of cooperation. Additionally, although there was no direct evidence showing psychological distance would influence the effects of shared experiences on cooperation, previous research revealed the influence of psychological distance on the effectiveness of shared experience. Research showed sharing an experience can amplify that experience, which was termed as amplification of shared experience. However, the experience would be amplified only for psychologically proximate others but not for distant ones [32]. It would be valuable to explore the findings of our study by introducing psychological distance in future studies.

## 5. Conclusions

Conflicts between the effects of positive and negative experiences existed in prior studies. The findings clarify how positive/negative experience affect cooperation and reconcile the conflicts. Through two experiments, our study found that positive/negative experience does not directly predict levels of cooperative behavior, and sharedness is an important moderating variable influencing the effect of positive/negative experiences on cooperation. Only when shared, a negative experience induces more cooperative behavior than a positive experience. When unshared, the effect of positive/negative experience on cooperative behavior shows no significant difference.

## Figures and Tables

**Table 1:** Payoff schedule for the economic game.

Number the Participant Choose	Lowest Number Chosen among the Group						
	1	2	3	4	5	6	7
1	¥4.20						
2	¥3.60	¥4.80					
3	¥3.00	¥4.20	¥5.40				
4	¥2.40	¥3.60	¥4.80	¥6.00			
5	¥1.80	¥3.00	¥4.20	¥5.40	¥6.60		
6	¥1.20	¥2.40	¥3.60	¥4.80	¥6.00	¥7.20	
7	¥0.60	¥1.80	¥3.00	¥4.20	¥5.40	¥6.60	¥7.80

Note: ¥ represents the currency symbol of CNY.

## Data Availability

The datasets generated for this study are available on request to the corresponding author.
